# Formula Compatibility Identification of Dachengqi Decoction Based on the Effects of Absorbed Components in Cerulein-Injured Pancreatic AR42J Cells

**DOI:** 10.1155/2016/3198549

**Published:** 2016-03-31

**Authors:** Yumei Zhang, Lin Zhu, Jia Wang, Jianlei Zhao, Xianlin Zhao, Hui Guo, Juan Li, Wenfu Tang

**Affiliations:** ^1^Sichuan Provincial Pancreatitis Center, Department of Integrative Medicine, West China Hospital, Sichuan University, Chengdu 610041, China; ^2^Department of Intensive Care Medicine, Sichuan Integrative Medicine Hospital, Chengdu 610041, China; ^3^Department of General Practice, Sichuan Provincial People's Hospital, Chengdu 610072, China; ^4^Department of Pharmacology, School of Preclinical and Forensic Medicine, West China Medical Center, Sichuan University, Chengdu 610041, China

## Abstract

*Objective*. To identify the herbal formula compatibility law based on the effects of the absorbed components from DCQD on the cerulein-injured AR42J cells.* Methods*. AR42J cells were pretreated for 30 min with or without the different concentrations of the absorbed components from DCQD individually or in combination or DCQD and coincubated with cerulein (10 nM) for a further 24 h. Cell viability, lactate dehydrogenase (LDH) release, and the levels of apoptosis and necrosis were measured.* Results*. Compared to DCQD, the individual or combination components partially protected cerulein-injured AR42J cells by increasing cell viability, reducing LDH release, and promoting apoptosis. Rhein, naringin, and honokiol were the main absorbed components from DCQD in cerulein-induced pancreatitis. Moreover, rhein in combination with naringin and honokiol had synergistic effects in protecting cerulein-injured AR42J cells and was better than the individual or the pairwise combination of the three components.* Conclusions*. The ten effective components from DCQD may elicit similar protective effects as DCQD on cerulein-induced pancreatitis. The principle of the formula compatibility of DCQD may be identified based on the effects of its absorbed components in cerulein-injured AR42J cells.

## 1. Introduction

Dachengqi decoction (DCQD) is traditional Chinese herbal decoction composed of* Radix et Rhizoma Rhei* (Dahuang),* Cortex Magnoliae Officinalis* (Houpo),* Fructus Aurantii Immaturus* (Zhishi), and* Natrii Sulfas* (Mangxiao). It has been used to treat acute pancreatitis (AP) for more than three decades in China [1]. Recent studies reported that DCQD could promote the gastrointestinal motility and inhibit the cytokine's activity and the inflammatory response in patients with AP [2, 3]. According to the prescription compatibility principle of traditional Chinese medicine (TCM) formulations, Dahuang is the principal drug in DCQD, while Mangxiao, Houpo, and Zhishi are assistant ingredients. Our previous studies found that good prescription compatibility in TCM could increase the level of rhein and affect its pharmacokinetics [4, 5]. Unfortunately, the related optimal prescription compatibility of the absorbed components from DCQD in AP remains unclear.

AP is one disease largely depending on the balance between apoptosis and necrosis in pancreatic acinar cells. The induction of apoptosis might be one self-protective factor protecting against acinar cell injury and reducing the severity of pancreatitis because of the release of variety intracellular zymogen which could reduce the inflammatory response [6, 7]. It has been reported that the induction of pancreatic acinar cell apoptosis by crambene protects mice against AP by the induction of anti-inflammatory pathways [8], whereas for necrosis, in which cell membrane integrity is lost in association with the release of digestive enzymes, lactate dehydrogenase (LDH) and inflammatory mediators can lead to local and systemic inflammatory response and damage [6, 9–11]. Furthermore, our previous studies found that DCQD could inhibit local and systematic inflammatory responses and alleviate pancreatic damage by regulating the necrosis-apoptosis switch of the pancreatic cells in AP [12]. The ten bioactive components of DCQD, namely, rhein, emodin, aloe-emodin, chrysophanol, rheochrysidin, naringin, naringenin, hesperidin, honokiol, and magnolol, were detected in the serum of rats and dogs [13, 14]. However, it is unclear whether these individual components or the related combination has the similar effects to DCQD in the treatment of AP. This study investigated the protective effect of the individual component and the related combination by testing cell viability, lactate dehydrogenase (LDH) release, and levels of apoptosis and necrosis in cerulein-injured pancreatic AR42J cells, to identify the herbal formula compatibility law of DCQD based on the effects of its absorbed components on cerulein-injured AR42J cells.

## 2. Materials and Methods

### 2.1. Materials

Rhein, emodin, aloe-emodin, chrysophanol, rheochrysidin, naringin, naringenin, hesperidin, honokiol, and magnolol were purchased from the Sichuan Academy of Chinese Medicine Sciences (Chengdu, China). Their purities were higher than 99%. Spray-dried Dahuang, Houpo, Zhishi, and Natrii Sulphas powders were purchased from Chengdu Green Herbal Pharmaceutical Co. Ltd. (Chengdu, China). The mean contents of the components from DCQD detected three times in our previous study were as follows: rhein, 0.86 mg/g; emodin, 2.48 mg/g; aloe-emodin, 1.73 mg/g; chrysophanol 0.55 mg/g, rheochrysidin, 2.61 mg/g; naringin, 3.83 mg/g; naringenin 4.16 mg/g; hesperidin, 11.06 mg/g; honokiol, 1.26 mg/g; magnolol, 1.11 mg/g [13]. The peak concentrations of these ten components in serum when rats were administered DCQD with 20 g/Kg·BW, as reported in the previous studies, are as follows: rhein, 365.67 ng/mL; emodin, 3.62 ng/mL; chrysophanol, 36.33 ng/mL; rheochrysidin, 1.83 ng/mL; aloe-emodin, 10.47 ng/mL; magnolol, 1.08 ng/mL; honokiol, 9.07 ng/mL; naringin, 42.83 ng/mL; hesperidin, 40.95 ng/mL; and naringenin, 22.67 ng/mL [12, 15]. The stock solutions of these components prepared at the reported peak serum concentrations were diluted 1/2, 1/4, 1/8, 1/16, and 1/32 in distilled water. Spray-dried powders were mixed with an equal amount of distilled water and reconstituted in dimethylsulfoxide (DMSO) to prepare a 50 mg/mL stock solution and kept in −20°C. The final DMSO concentration was less than 0.1% when the components were added to the cell culture media. The dosage required was calculated and samples were diluted and quantitatively analyzed by high-performance liquid chromatography (HPLC). Fetal bovine serum (FBS) was obtained from HyClone (Logan, UT). DMSO, cerulein, F12K medium, and 2′,7′-dichlorofluorescin diacetate(DCFH-DA) were obtained from Sigma (St. Louis, MO, USA).

### 2.2. Methods

#### 2.2.1. Cell Culture

The rat pancreatic acinar AR42J cells (ATCC, Rockville, MD, USA) were cultured in F12K medium containing 20% FBS, 100 U/mL penicillin, and 100 *μ*g/mL streptomycin under standard conditions (37°C and 5% CO_2_). All experiments were performed 24 h after cells were seeded into culture plates. AR42J cells were treated for 30 min with or without the selected dosage of the components and then coincubated with cerulein (10 nM) for a further 24 h.

#### 2.2.2. Cell Viability Assay

Cell survival was assessed using the WST viability assay kit containing WST-8 (2-(2-methoxy-4-nitrophenyl)-3-(4-nitrophenyl)-5-(2,4-disulfophenyl)-2H-tetrazolium, monosodium salt) according to the manufacturer's protocol (Roche, Basel, Switzerland). AR42J cells were plated into 96-well plates at a density of 2 × 10^4^ cells/well. After incubation for 24 h, cells were pretreated with various concentrations of the components and coincubated with cerulein for a further 24 h. After cerulein is added, cell viability was detected by the kit at 2 h, 4 h, 6 h, 12 h, and 24 h. WST-8 solution (0.5 mg/mL) was added to each well and cells were incubated at 5% CO_2_ and 37°C for 2 h. Cell viability was determined by the different absorbance at wavelengths of 630 nm. The relative cell viability rate was calculated using the following formula: cell viability rate (%) = 100%  ×  mean absorbance of cells in the treated group/mean absorbance of cells in control group.

#### 2.2.3. LDH Assay

Necrotic cell death was assessed by the release of LDH from the cytosol of damaged cells into the supernatant using the LDH Cytotoxicity Detection Kit (Nanjing Jiancheng Bioengineering Institute, Nanjing, China) at various time points (0–24 h) according to the manufacturer's instructions. The percentage of total cellular LDH released was determined using the following equation: LDH release (%) = total extracellular LDH activity at the given time point × 100/total LDH activity at the given time point.

#### 2.2.4. Apoptosis Assay

Cells were stained with the Annexin V-FITC Apoptosis Detection Kit (Nanjing Kaiji, Nanjing, China) according to the manufacturer's instructions to detect early apoptotic cells (annexin V^+^ and propidium iodide (PI)^−^) and necrotic or late apoptotic cells (annexin V^+^ and PI^+^) by flow cytometry. Briefly, AR42J cells were treated with or without various components for 30 min and then stimulated with cerulein (10 nM) for 24 h. Cells were collected and resuspended in culture medium at a density of 1 × 10^6^ cells/mL and stained with 5 *μ*L of annexin V-FITC and 5 *μ*L PI prepared in 300 *μ*L binding buffer (10 mM HEPES, pH 7.4, 140 mM NaOH, and 2.5 mM CaCl_2_) according to the manufacturer's instructions for 15 min at room temperature in the dark. Cells were analyzed by flow cytometry (FACScan, Becton Dickinson, USA).

#### 2.2.5. Statistical Analysis

Statistical analyses were performed using the PEMS3.1 statistics program. All data represented at least three independent experiments and were expressed as the mean ± standard deviation (mean ± SD). One-way repeated-measures ANOVA (followed by multiple pairwise comparisons using the Student-Newman-Keuls procedure) was used to analyze differences between experimental and control groups. *p* < 0.05 was regarded as statistically significant.

## 3. Results

### 3.1. Treatment with the Ten Components Individually from DCQD Increased Cell Viability in Cerulein-Injured AR42J Cells

**Figure 1:**
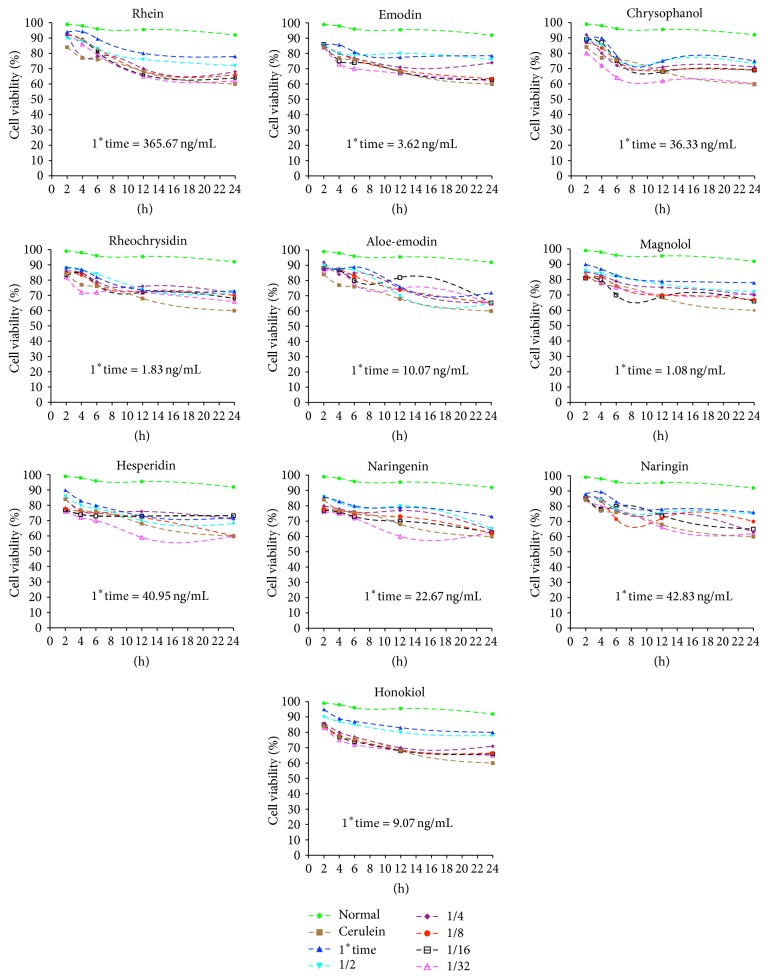
The effects of the ten absorbed components from DCQD on the cerulein-induced necrosis of AR42J cells. Cells were pretreated with various concentrations (1^*∗*^time, 1/32, 1/16, 1/8, 1/4, or 1/2) of each component for 30 min and then coincubated with 10 nM cerulein for 24 h. After cerulein is added, cell viability is examined by WST-8 assay at 2 h, 4 h, 6 h, 12 h, and 24 h. 1^*∗*^time concentration means the peak concentration of the components in serum detected by our previous study.

To examine the effects of various components on cell viability, pancreatic acinar AR42J cells were pretreated with six concentrations (undiluted or diluted 1/32, 1/16, 1/8, 1/4, or 1/2) of each component and cerulein for 24 h. Cell viability was markedly decreased in cerulein-treatment group. Each component had a protective effect on cerulein-induced cell death in a concentration-dependent manner, and the most proper treatment concentration was undiluted one, the peak concentration (Figure 1).

### 3.2. Treatment with the Ten Components from DCQD Individually Reduced Cell Death and LDH Release in Cerulein-Injured AR42J Cells (Table 1)

The effects of various components of DCQD on the viability of AR42J cells and their LDH release upon cerulein treatment were assessed. Viability was highest among rhein-treated cells than those treated with emodin, aloe-emodin, chrysophanol, or rheochrysidin from Dahuang (Table 1). LDH release was lowest among rhein-treated cells than those treated with any other component from Dahuang (*p* < 0.05). In comparison with cells treated with hesperidin or naringenin from Zhishi, naringin-treated cells exhibited a higher viability (*p* < 0.05) and lower LDH release (*p* < 0.05). Cell viability was higher and LDH release was lower among honokiol-treated cells than that among magnolol-treated cells (*p* < 0.05). Rhein, naringin, and honokiol may be the main absorbed components of Dahuang, Zhishi, and Houpo, respectively.

### 3.3. Treatment with Combination Components from DCQD Reduced Cell Death and LDH Release in Cerulein-Induced AR42J Cells

According to the aforementioned results, rhein is a major absorbed component from Dahuang and protects against cerulein-injured AR42J cells and reduces LDH release. Similarly, naringin and honokiol are considered to be the major absorbed components of Zhishi and Houpo, respectively. What is more, the most effective concentration was the peak concentration according to our first result; therefore, we explored whether treatment with combinations of the three major components in peak concentrations or with the mixture of the ten components induced similar effects to DCQD. The levels of cerulein-induced AR42J cell death and LDH release were measured after pretreatment with the various combinations of components (Table 2). Compared to untreated cells, the cell viability was significantly reduced among the cells treated with cerulein. However, this decrease in the cell viability was significantly prevented among the cells pretreated with rhein, honokiol, naringin, rhein plus honokiol, rhein plus naringin, naringin plus honokiol, rhein plus honokiol and naringin, the mixture of the ten components, or DCQD. The cell viability was higher among rhein-treated cells than honokiol plus naringin-treated cells. The cell viability increased significantly when the cells were treated with rhein plus honokiol, rhein plus honokiol and naringin, or the mixture of the ten components. However, the cell viability was higher among the cells treated with DCQD than among those treated with any of the components, either individually or in combination, including those treated with all ten components. LDH release was relatively low in the untreated cells and was significantly increased by cerulein treatment. Pretreatment with rhein, honokiol, naringin, rhein plus honokiol, rhein plus naringin, naringin plus honokiol, rhein plus honokiol and naringin, the mixture of the ten components, or DCQD significantly reduced LDH release. LDH release was lower among the cells treated with DCQD than among those treated with any of the components, either individually or in combination. LDH release was lower among the cells treated with rhein than among those treated with honokiol and/or naringin. All these results showed that the components, either individually or in combination, including the mixture of ten components demonstrated some efficacy of the prescription of DCQD.

### 3.4. Treatment Individually or in Combination with the Components of Rhein, Honokiol, and Naringin from DCQD Showed a Synergistic Effect on the Apoptosis-Necrosis Cellular Switch in Cerulein-Induced AR42J Cells

**Figure 2:**
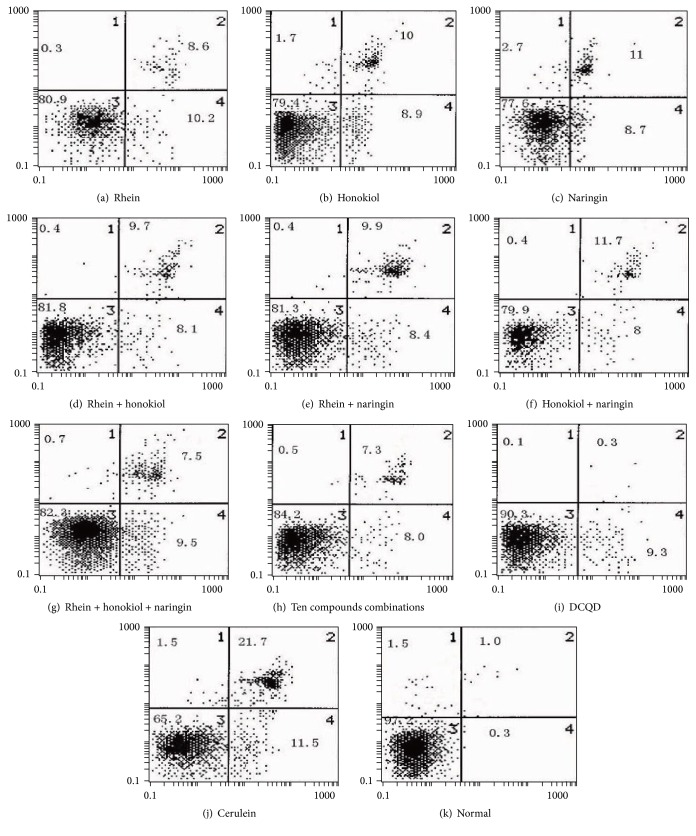
The synergistic effects of rhein, honokiol, and naringin from DCQD on the apoptosis-necrosis cellular switch in cerulein-induced AR42J cells. Cells were pretreated with or without the three major components with the peak concentrations individually or in combination for 30 min and then coincubated with 10 nM cerulein for 24 h. Annexin V/PI staining was performed and flow cytometry analyses were used. Each panel is divided into four regions: viable cells (annexin V^−^/propidium iodide (PI)^−^) are located in the lower left quadrant, early apoptotic cells (annexin V^+^/PI^−^) in the lower right quadrant, late apoptotic and necrotic cells (annexin V^+^/PI^+^) in the upper right quadrant, and primary necrotic cells (annexin V^−^/PI^+^) in the upper left quadrant.

Our previous study found that DCQD could regulate the apoptosis-necrosis switch of pancreatic acinar cells in rats with AP or in isolated cells. Annexin V/PI staining was performed to assess whether the level of apoptosis differed among samples treated with the three major bioactive components of DCQD, individually or in combination. Annexin V^−^/PI^−^ cells were regarded as healthy, annexin V^+^/PI^−^ cells were regarded as early apoptotic, and annexin V^+^/PI^+^ cells were regarded as necrotic or late apoptotic (Figure 2). Flow cytometry analyses suggested an extremely low level of cell death in untreated samples, and this was markedly increased following treatment with cerulein for 6 h. The individual treatment, the pairwise combination, or all the three components of rhein, honokiol, and naringin could increase the rate of apoptosis in AR42J cells, but the percentage of apoptotic cells treated with all the three components of rhein, honokiol, and naringin was significantly highest among all the six treatment groups (*p* < 0.05) (Table 3), which showed a synergistic effect on the apoptosis-necrosis cellular switch in cerulein-induced AR42J cells.

## 4. Discussion

This study identified the protective effect of individual component and related combination of the components from DCQD in dose-dependent and time-dependent manner on cerulein-induced AR42J cells. Among the ten components, pretreatment with rhein of the peak serum concentration in cerulein-induced AR42J cells showed the strongest protective effect among the components from Dahuang, such as cell viability and LDH release, on injured AR42J cells. Similarly, naringin and honokiol showed similar protective effect in injured AR42J cells. Rhein, naringin, and honokiol may be the major effect components from DCQD in treatment of AP in vitro. All these results showed that the components, either individually or in combination, have some efficacy of the prescription of DCQD. It is similar to the study of Guan-Xin-Er-Hao formula [16] which indicated that the combination of three absorbed bioactive components (ferulic acid, tanshinol, and hydroxysafflor yellow A) is similar to its formula in reducing infarct size of acute myocardial infarction (AMI) in rats. The study of Ju-Zhi-Jiang-Tang (JZJT) [17] showed that the two major active constituents (nobiletin and tangeretin) can significantly exert anti-inflammatory effects representing the efficacy of the formula. Another research [18] suggested that claycosin and formononetin from Yu-Ping-Feng-San (YPFS) can reduce allergic inflammation similar to the effect of YPFS in vivo and in vitro. Similarly, the major effective components of Shaoyao-Gancao decoction [19], Bu-Shen-Yi-Qi Fang [20, 21], Zhi-zi-chi decoction [22], Yin-Chen-Hao-Tang [23], and so forth represent part efficacy of formula. Therefore, many major absorbed components of herbs individually or in combination partly have efficacy of formula.

Interestingly, combining rhein with honokiol, but not with naringin, had an additive protective effect. Pretreatment with naringin and honokiol elicited similar effects on proapoptosis as treatment with rhein. Pretreatment with the three components showed more positive effects than treatment with each of them alone. With respect to the regulation of the necrosis-apoptosis switch, treatment with rhein, naringin, honokiol, rhein plus naringin, rhein plus honokiol, naringin plus honokiol, rhein plus naringin plus honokiol, all ten components, or whole DCQD promoted injured cell apoptosis. The percentage of apoptotic cells was higher among cells treated with rhein plus naringin plus honokiol and all ten components than those treated with rhein alone (*p* < 0.05) (Table 3). Moreover, the percentage of apoptotic cells was highest among cells treated with DCQD (*p* < 0.05). This identified the herbal formula compatibility based on the synergistic effects of rhein, honokiol, and naringin from DCQD in vitro study on the pancreatic AR42J cells. Many researches have confirmed the compatibility principle of formula or herbs via the combination of the major effective components in vivo or in vitro, especially the additive or synergistic effects of the absorbed components. The study of Sini decoction [24] proved that the major active ingredients (the total alkaloids, total gingerols, total flavones, and total saponins) were more effective than formulas formed by any one or two of the three individual components. In another study [25], amygdalin and hydroxysafflor yellow A, main components of Taoren-Honghua (TH) herb pair, are responsible for the main curative effects of TH and usually have synergetic effects, such as decreasing plasma viscosity and platelet aggregation percentage. What is more, total coumarins and volatile oil, as the two main components of* Radix Angelicae dahuricae*, can improve the intestinal absorption of baicalin extracted from* Scutellaria baicalensis Georgi* and have synergistic action in the enhanced absorption of baicalin, where* Angelicae dahuricae* and* Scutellaria baicalensis Georgi* is one herb pair, which has clarified the compatibility principles of herb pairs [26]. In our study, rhein, naringin, and honokiol had synergistic effects, and naringin plus honokiol had an additive effect on rhein in proapoptosis, which proved the compatibility principle of herbs.

In this study, treatment with each of the ten absorbed components of DCQD could elicit partial effects as DCQD treatment on cerulein-induced pancreatitis. Rhein, naringin, and honokiol may be the major effect components of DCQD in treatment of AP in vitro. Moreover, treatment with combinations of these three components elicited better synergistic effects on cerulein-induced pancreatic acinar cells than individual components treatment, which help to identify the herbal formula compatibility law of DCQD based on the effects of its absorbed components on cerulean-injured AR42J cells. Further studies are needed to determine the optimal ratio of rhein, naringin, and honokiol for protection against acinar cell death and to elucidate the underlying mechanism.

## Figures and Tables

**Table 1:** The effects of the absorbed components from DCQD individually on cerulein-induced AR42J cell death and LDH release.

Component	Cell viability (%)	LDH release (%)
Rhein	76.02 ± 1.32	20.73 ± 0.78
Emodin	73.05 ± 1.73	24.15 ± 0.67^*∗*^
Chrysophanol	73.05 ± 1.73	22.44 ± 0.47^*∗*^
Rheochrysidin	72.76 ± 2.04	24.87 ± 1.03^*∗*^
Aloe-emodin	71.38 ± 1.58^*∗*^	25.73 ± 0.84^*∗*^
Naringin	72.48 ± 1.02	26.82 ± 0.96
Hesperidin	69.60 ± 1.64^◆^	29.63 ± 1.21^◆^
Naringenin	68.40 ± 1.77^◆^	33.04 ± 0.57^◆^
Honokiol	75.04 ± 1.64	21.71 ± 0.66
Magnolol	73.35 ± 2.11^▲^	22.68 ± 0.75^▲^
Cerulein alone	63.47 ± 1.03	40.24 ± 2.03
Normal	92.30 ± 1.32^*∗*^	5.31 ± 0.23^*∗*^

Rhein, emodin, chrysophanol, rheochrysidin and aloe-emodin are from Dahuang, naringenin, naringin and hesperidin are from Zhishi, and magnolol and honokiol are from Houpo. LDH = lactate dehydrogenase. Cells were pretreated with the ten components with the peak concentrations for 30 min and then coincubated with 10 nM cerulein for 24 h. After cerulein added, cell viability examined by WST-8 assay. Necrotic cell death was assessed by the release of LDH from the cytosol of damaged cells into the supernatant using the LDH Cytotoxicity Detection Kit. The results are mean ± SD. ^*∗*^ *p* < 0.05 versus rhein-treated group, ^◆^ *p* < 0.05 versus naringin-treated group; ^▲^ *p* < 0.05 versus honokiol-treated group.

**Table 2:** The effects of rhein, honokiol, and naringin individually or in combination on cerulein-induced AR42J cell death and LDH release.

Component	Cell viability (%)	LDH release (%)
R	72.96 ± 1.38^*∗*◆^	22.57 ± 0.79^*∗*◆^
H	71.02 ± 0.79^*∗*▲◆^	24.63 ± 0.59^*∗*▲◆^
N	70.40 ± 1.50^*∗*▲◆^	26.83 ± 1.38^*∗*▲◆^
R plus H	74.43 ± 2.13^*∗*▲◆^	21.09 ± 2.03^*∗*▲◆^
R plus N	72.46 ± 1.34^*∗*◆^	22.04 ± 0.46^*∗*◆^
N plus H	72.15 ± 0.67^*∗*◆^	24.39 ± 0.73^*∗*▲◆^
R plus H plus N	76.02 ± 0.93^*∗*▲◆^	20.73 ± 1.37^*∗*▲◆^
Ten components	78.95 ± 1.88^*∗*▲◆^	17.07 ± 0.87^*∗*▲◆^
DCQD	80.44 ± 2.38^*∗*▲^	15.85 ± 0.45^*∗*▲^
Cerulein alone	63.47 ± 1.03	40.24 ± 2.03
Normal	92.30 ± 1.32^*∗*^	5.31 ± 0.23^*∗*^

R = rhein, H = honokiol, N = naringin, DCQD = Dachengqi decoction, and LDH = lactate dehydrogenase. Cells were pretreated with the components with the peak concentrations for 30 min and then coincubated with 10 nM cerulein for 24 h. After cerulein is added, cell viability is examined by WST-8 assay. Necrotic cell death was assessed by the release of LDH from the cytosol of damaged cells into the supernatant using the LDH Cytotoxicity Detection Kit. The results are mean ± SD. ^*∗*^ *p* < 0.05 versus cerulein alone-treated group, ^▲^ *p* < 0.05 versus rhein-treated group, and ^◆^ *p* < 0.05 versus DCQD-treated group.

**Table 3:** The proapoptotic effects of rhein, honokiol, and naringin from DCQD on cerulein-induced AR42J cells.

Component	Apoptotic cells (%)
R	43.62 ± 2.38^#◆^
H	47.09 ± 2.54^#◆^
N	44.16 ± 1.84^#◆^
R plus H	45.51 ± 2.31^#◆^
R plus N	38.53 ± 1.67^#▲◆^
N plus H	40.61 ± 2.37^#◆^
R plus H plus N	55.88 ± 1.94^#▲◆^
Ten components	52.29 ± 3.17^#▲◆^
DCQD	96.87 ± 2.42^#▲^
Cerulein alone	34.64 ± 2.30^*∗*^
Normal	23.08 ± 2.32^#^

R = rhein, H = honokiol, N = naringin, and DCQD = Dachengqi decoction. Ten components = the mixture of the ten components from DCQD. AR42J cells were pretreated with or without rhein, honokiol, and naringin with the peak concentrations individually or in combination for 30 min and then stimulated with cerulein (10 nM) for 24 h. Cells were stained with the Annexin V-FITC Apoptosis Detection Kit and analyzed by flow cytometry. The results are mean ± SD. ^*∗*^ *p* < 0.05 versus normal group; ^▲^ *p* < 0.05 versus rhein-treated group; ^◆^ *p* < 0.05 versus DCQD-treated group; ^#^ *p* < 0.05 versus cerulein alone-treated group.

## Abbreviations

DCQD: Dachengqi decoction
AP: Acute pancreatitis
LDH: Lactate dehydrogenase
DMSO: Dimethylsulfoxide
HPLC: High-performance liquid chromatography
FBS: Fetal bovine serum
DCFH-DA: 2′,7′-Dichlorofluorescin diacetate
AMI: Acute myocardial infarction.
